# Effect of Methylation Status of lncRNA-MALAT1 and MicroRNA-146a on Pulmonary Function and Expression Level of COX2 in Patients With Chronic Obstructive Pulmonary Disease

**DOI:** 10.3389/fcell.2021.667624

**Published:** 2021-09-08

**Authors:** Li Sun, Aiqun Xu, Min Li, Xingyuan Xia, Pulin Li, Rui Han, Guanghe Fei, Sijing Zhou, Ran Wang

**Affiliations:** ^1^Department of Respiratory and Critical Care Medicine, The First Affiliated Hospital of Anhui Medical University, Hefei, China; ^2^Department of General Medicine, Hefei Second People’s Hospital, Hefei, China; ^3^Department of Oncology, The First Affiliated Hospital of Anhui Medical University, Hefei, China; ^4^Hefei Third Clinical College of Anhui Medical University, Hefei, China; ^5^Hefei Prevention and Treatment Center for Occupational Diseases, Hefei, China

**Keywords:** COPD, MALAT1, miRNA, COX2, methylation

## Abstract

This study aimed to investigate the role of methylation of MALAT1 and miR-146a in the pathogenesis of chronic obstructive pulmonary disease (COPD). COPD patients were grouped according to their methylation status of MALAT1 and miR-146a promoters, and we found that forced vital capacity, volume that has been exhaled at the end of the first second of forced expiration, and diffusion capacity for carbon monoxide were the highest in the MALAT1 HYPO + miR-146a HYPER group and lowest in the MALAT1 HYPER + miR-146a HYPO group, and COPD patients with hypermethylated MALAT1 showed lower expression of MALAT1 than that in the COPD patients with hypomethylated MALAT1. Meanwhile, miR-146a was the most significantly upregulated in the MALAT1 HYPER + miR-146a HYPO group and the most significantly downregulated in the MALAT1 HYPO + miR-146a HYPER group. Both prostaglandin E_1_ and cyclooxygenase 2 (COX2) expression were the highest in the MALAT1 HYPO + miR-146a HYPER group and the lowest in the MALAT1 HYPER + miR-146a HYPO group. In conclusion, our results established a MALAT1/miR-146a/COX2 signaling axis. The overexpression of MALAT1 could increase the expression of COX2 by inhibiting the expression of miR-146a, thus affecting the pulmonary function of COPD patients.

## Introduction

Chronic obstructive pulmonary disease (COPD) is actually the fourth leading cause of mortality around the world (Vogelmeier et al., 2017). Smoking is the leading danger of COPD, while the cessation of cigarette smoking in the beginning of COPD might slow down or even switch back its course in the reduction of lung functions (Damkjær et al., 2021). COPD is actually featured via chronic inflammation in the lungs, which participates in significant steps in the advancement of the disease (Vogelmeier et al., 2017; Chan et al., 2019).

Long non-coding RNAs (lncRNAs) possess unique functions in a myriad of cell processes including the potential to inhibit the expression of neighboring protein-coding genes or the control of protein activities (Willingham et al., 2005; Yu et al., 2008; Zhao et al., 2008). Metastasis-associated lung adenocarcinoma transcript 1 (MALAT-1) is an lncRNA initially shown to become overexpressed in early non-small cell lung carcinoma and was used as a marker for the prognosis of metastasis (Ji et al., 2003; Hutchinson et al., 2007). MALAT1 could be processed to produce an ncRNA, as well as a tRNA-like cytoplasmic RNA (Wilusz et al., 2008). It has been presented that MALAT1 targets miR-146a in cells. Furthermore, the protective impacts of MALAT1 on the apoptosis, as well as viability of chondrocytes exposed to lipopolysaccharide, were partially inversed via miR-146a downregulation (Li G. Q. et al., 2019). As a regulator in both adaptive and innate immunity, miR-146a was actually related to both the onset of lymphoma and the suppression of tumors, such as in T cells infected by human T-cell leukemia virus 1 (Monticelli et al., 2005; Taganov et al., 2006; Peveling-Oberhag et al., 2012). On top of that, in lymphoma samples fixed in formalin and embedded in paraffin that were from subjects with diffuse large B-cell lymphoma, the expression of miR-146a possessed a prognostic function: reduced miR-146a expression was actually related to a much higher rate of total remission (Zhong et al., 2012). On the other hand, in patients with natural killer/T-cell lymphoma (NKTL), miR-146 functions as a tumor suppressor, and reduced miR-146a expression was linked to poor prognosis, whereas miR-146a overexpression prevented the growth of NKTL cells (Paik et al., 2011).

Cyclooxygenase 2 (COX2) and prostaglandin E_2_ (PGE2), the enzymatic product of COX2, play an essential role in disease pathogenesis (Greenhough et al., 2009). COX2 is actually associated with the transformation of arachidonic acid into prostaglandin H_2_, a compound that is later converted to prostacyclin PGI2; prostaglandins PGE2, PGD2, and PGF2α; and thromboxane A_2_ (Asting et al., 2011; Allaj et al., 2013; Vogel et al., 2014). Particularly, after stimulation by cytokines, COX2 expression in COPD was actually increased. However, the elevation was considerably higher in COPD cells. This distinguishes from the absence of changes in COX1 expression after interleukin 1b (IL-1b) and tumor necrosis factor (TNF-α) stimulation, after which the rise in basal expression was completely triggered by COX1, whereas the notable boost in COX activities of COPD fibroblasts was triggered by COX2, suggesting that COX1 might make up for the boosted basal synthesis of PGE2. However, the raised PGE2 synthesis upon IL-1b as well as TNF-α stimulation is triggered by COX2 (Sato et al., 2010).

It has been reported that the methylation status of MALAT1 and miR-146a promoters is associated with their expression (Szenthe et al., 2013; Guo et al., 2015). Furthermore, MALAT regulates the expression of miR-146a via sponging, and a previous study of our research group showed that the expression of COX2 and its product, PGE, is controlled by miR-146a (Wang et al., 2015; Li G. Q. et al., 2019). In this study, we collected clinical samples from COPD subjects to investigate the effect of MALAT1 and miR-146a methylation on the severity of COPD, pulmonary function, and COX2 expression.

## Materials and Methods

### Patient Recruitment

Chronic obstructive pulmonary disease is a type of lung disorder identified by severe and persistent obstruction of the airflow in the lungs that hampers ordinary breathing, and the symptoms of COPD cannot be completely reversed (as defined by the World Health Organization). To investigate the role of methylation of MALAT1 and miR-146a in the pathogenesis of COPD, a total of 168 COPD patients were enrolled in this study. Based on the status of methylation of MALAT1 and miR-146a promoters in these patients, they were assigned to four different groups: first, the participants were grouped based on the methylation status of MALAT1 as hypermethylation (above median, *n* = 84) and hypomethylation (below median, *n* = 84). Each of the two above MALAT1 groups was further divided into two groups based on the methylation status of miR-146a as hypermethylation (above median, *n* = 42) and hypomethylation (below median, *n* = 42) in each group. Thus, the four groups were as follows: MALAT1 HYPERMETHYLATED + miR-146a HYPOMETHYLATED group (termed as MALAT1 HYPER + miR-146a HYPO group, *n* = 42), MALAT1 HYPERMETHYLATED + miR-146a HYPERMETHYLATED group (termed as MALAT1 HYPER + miR-146a HYPER group, *n* = 42), MALAT1 HYPOMETHYLATED + miR-146a HYPOMETHYLATED group (termed as MALAT1 HYPO + miR-146a HYPO group, *n* = 42), and MALAT1 HYPOMETHYLATED + miR-146a HYPERMETHYLATED group (termed as MALAT1 HYPO + miR-146a HYPER group, *n* = 42). Patient information, including their age, sex, body height, weight, smoking status, stage according to the Global Initiative for Chronic Obstructive Lung Disease (GOLD) (Halpin et al., 2021), forced vital capacity (FVC), forced expiratory volume (FEV), and diffusion capacity for carbon monoxide (DLCO), was collected and compared among the four groups. In addition, peripheral blood samples and peripheral blood mononuclear cell (PBMC) samples were also collected from each patient to examine the expression of MALAT1, miR-146a, and PGE1 in different groups. All participants of this study were Chinese people of Han ethnicity. This study was reviewed and approved by the ethical review committee of Anhui Medical University.

### Evaluation of Pulmonary Functions

The stages of COPD of the study participants were defined, depending on the standards shown in GOLD: volume that has been exhaled at the end of the first second of forced expiration (FEV_1_) in 1 s of <30%, GOLD4 as well as “very severe”; 30% ≤ FEV_1_ < 50%, GOLD3 as well as “severe”; 50% ≤ FEV_1_ < 80%, GOLD2 as well as “moderate”; and FEV_1_ > 80%, GOLD1 as well as “mild.” To evaluate pulmonary functions, the spirometry test was carried out to determine the FVC and the FEV_1_ by utilizing a spirometer based on the protocol provided by the manufacturer.

### RNA Isolation and Real-Time Polymerase Chain Reaction

The experiments were performed as previously described (Wang et al., 2018; Zhou et al., 2018a). Total RNA was extracted from tissue and cell samples by using a one-step Trizol RNA isolation assay kit (Invitrogen, Carlsbad, CA, United States) based on the protocol provided by the assay kit manufacturer. Then, the cDNA of miR-146a, MALAT1, as well as COX2 mRNA, was reverse transcribed from the isolated total RNA by using a reverse transcriptase reagent kit (Applied Biosystems, Foster City, CA, United States) based on the protocol provided by the reagent manufacturer. In the next step, real-time polymerase chain reaction (PCR) was carried out to determine the expression of miR-146a, MALAT1, and COX2 mRNA in each sample using a TaqMan SYBR Green Master Mix assay kit (Thermo Fisher Scientific, Waltham, MA, United States) on a PRISM 7900 real-time PCR machine (Applied Biosystems, Foster City, CA, United States) based on the protocols provided by the manufacturers of both the reagent kit and the real-time PCR machine. Finally, the relative expression of miR-146a (forward: 5′- GAGAACTGAATTCCATGG-3′; reverse: 5′-GAACATGTCTGCGTATCTC-3′), MALAT1 (forward: 5′-TCTGCAGGGACTACAGCAAG-3′; reverse: 5′-TCACATTGGTGAATCCGTCT-3′) and COX2 mRNA (forward: 5′-CGGTGAAACTCTGGCTAGACAG-3′; reverse: 5′- GCAAACCGTAGATGCTCAGGGA-3′) in each sample was calculated by using the 2^−(ΔΔ*Ct*)^ method, and U6 and GAPDH were used as the internal control for gene expression normalization.

### Cell Culture and Transfection

Human (hPASMCs) and rat pulmonary artery smooth muscle cells (rPASMCs) were isolated as previously described (Wang et al., 2015; Zhou et al., 2018b, 2019). The culture conditions of hPASMCs and rPASMCs were >95% humidity, 37°C, and 5% CO_2_. The cells were cultured in ordinary Dulbecco modified eagle medium (Gibco, Thermo Fisher Scientific, Waltham, MA, United States) added with 10% fetal bovine serum and suitable antibiotics (Gibco, Thermo Fisher Scientific, Waltham, MA, United States). To study the effects of MALAT1, the cells were divided into two sets of groups, with each subset of the groups further divided into two subgroups. In the first set of subgroups, hPASMCs and rPASMCs were divided into group 1 [NC (hPASMCs and rPASMCs treated with an empty vector)] and group 2 [pcDNA-MALAT1 (hPASMCs and rPASMCs treated with MALAT1 vector)]. In the second set of subgroups, hPASMCs and rPASMCs were divided into group 1 [NC (hPASMCs and rPASMCs treated with a scramble control siRNA)] and group 2 [MALAT1 siRNA (hPASMCs and rPASMCs treated with MALAT1 siRNA)]. For transfection experiments, miR-146a mimics and inhibitors were synthesized by RiboBio (Guangzhou, China). The sequences for the miR-146a mimics, inhibitor, and negative control were as follows: miR-146a mimics (5′-UGAGAACUGAAUUCCAUGGGUU-3′), miR-146a inhibitor (5′-AACCCAUGGAAUUCAGUUCUCA-3′), and negative control (5′-UUGUACUACACAAAAGUACUG-3′). The transfection was done by using Lipofectamine 2000 (Invitrogen) based on the protocol provided by the manufacturer. At 48 h after the transfection was started, the transfected cells were collected for gene expression assays.

### Vector Construction, Mutagenesis, and Luciferase Assay

As indicated by our computational analysis, a putative binding site of miR-146a was identified in MALAT1. Therefore, to study the regulatory relationship between MALAT1 and miR-146a expression, the wild-type promoter of MALAT1 was amplified by PCR and cloned into a pcDNA3.1 vector (Promega, Madison, WI) that contained a firefly luciferase reporter gene. The resulting vector was termed *wild-type MALAT1 plasmid*. At the same time, site-directed mutagenesis was carried out in the miR-146a binding site of wild-type MALAT1 promoter by using a site-directed mutagenesis assay kit (Stratagene, San Diego, CA, United States) based on the protocol provided by the kit manufacturer to generate the mutant-type MALAT1 promoter, which was also cloned into a pcDNA3.1 vector, and the resulting vector was termed *mutant type MALAT1 plasmid*. In the next step, hPASMCs and rPASMCs were co-transfected with the wild-type or mutant-type MALAT1 promoter in conjunction with miR-146a mimics or a negative control miRNA. At 24 h after the transfection was started, the luciferase activity in transfected cells was evaluated by using a dual luciferase reporter gene assay kit (Promega) on a TD-20/20 luminometer (Turner Biosystems, Sunnyvale, CA, United States) based on the general protocols provided by the manufacturers of both the reporter gene assay kit and the luminometer (Zhou et al., 2020; Zhu et al., 2020).

### Western Blot Analysis

The experiments were performed as previously described (Wang et al., 2011; Ding et al., 2017). The protein content was extracted from tissue and cell samples by using a RIPA lysis buffer (Invitrogen, Waltham, MA, United States) based on the protocol provided by the assay kit manufacturer. In the next step, the concentration of protein lysate was quantitatively evaluated by using a BCA protein assay kit (Thermo Fisher Scientific, Waltham, MA, United States) based on the protocol provided by the assay kit manufacturer, and an equal amount of the protein lysate from each sample was separated on a 10% sodium dodecyl sulfate–polyacrylamide gel. Then, the separated proteins were blotted to a polyvinylidene fluoride membrane, which was then blocked with 5% serum and incubated in sequence with primary anti-COX2 antibody, as well as horseradish peroxidase–conjugated secondary antibody (Abcam, Cambridge, CA, United States) based on the protocol provided by the antibody manufacturer. The signal of protein band was detected by utilizing an ECL chemofluorescence reagent (Pierce, Rockford, IL, United States) based on the protocol provided by the reagent manufacturer to calculate the relative expression of COX2 protein in each sample using the expression of β-actin protein as the control.

### Enzyme-Linked Immunosorbent Assay

The experiments were performed as previously described (Wang et al., 2012, 2014). The serum concentration of PGE1 was assessed by making use of an enzyme-linked immunosorbent assay (ELISA) (IDS, Fountain Hills, AZ, United States) based on the protocol provided by the assay kit manufacturer.

### Bisulfite Sequencing

To determine the status of methylation of MALAT1 and miR-146a promoters in each collected sample, DNA extracted from the samples was modified first with bisulfite (Zymo Research, Orange, CA, United States) based on the protocol provided by the reagent manufacturer and then subjected to PCR sequencing on an ABI 3700 sequencer (Applied Biosystems, Waltham, MA, United States) based on the protocol provided by the equipment manufacturer.

### Statistical Analysis

All experiments were repeated at least three times. Results are shown as mean ± standard deviations. All statistical analyses were carried out by utilizing SPSS software version 19 (IBM, Armonk, NY, United States). *p* < 0.05 was considered statistically significant. The internal-group comparisons were made using Student *t* test, and the multigroup comparisons were made using one-way analysis of variance (Tukey test as *post hoc* test).

## Results

### Characteristics of COPD Patients

A total of 168 COPD patients were recruited during May 2015 to October 2018 and were further assigned to different groups according to their methylation of MALAT1 and miR-146a promoters, that is, the MALAT1 HYPERMETHYLATED + miR-146a HYPOMETHYLATED (as MALAT1 HYPER + miR-146a HYPO, *n* = 42) group, MALAT1 HYPERMETHYLATED + miR-146a HYPERMETHYLATED (as MALAT1 HYPER + miR-146a HYPER, *n* = 42) group, MALAT1 HYPOMETHYLATED + miR-146a HYPOMETHYLATED (as MALAT1 HYPO + miR-146a HYPO, *n* = 42) group, and the MALAT1 HYPOMETHYLATED + miR-146a HYPERMETHYLATED (as MALAT1 HYPO + miR-146a HYPER, *n* = 42) group. Patient information was collected and is listed in Table 1. The data in Table 1 revealed no obvious differences among different groups in terms of age, sex, body height, weight, and smoking history. However, FVC, FEV_1_, and DLCO were all highest in the MALAT1 HYPO + miR-146a HYPER group and lowest in the MALAT1 HYPER + miR-146a HYPO group.

**TABLE 1:** Demographic and clinicopathological characteristics of the participants in this study.

Characteristics	MALAT1 Hypermethylated + miR-146a Hypomethylated (*n* = 42)	MALAT1 Hypermethylated + miR-146a Hypermethylated (*n* = 42)	MALAT1 Hypomethylated + miR-146a Hypomethylated (*n* = 42)	MALAT1 Hypomethylated + miR-146a Hypermethylated (*n* = 42)	*P* value
Age, years	63.1 ± 5.4	62.6 ± 6.6	64.6 ± 8.3	62.6 ± 3.8	0.521
Gender (male/female)	39/3	37/5	39/3	40/2	0.666
Height, cm	167.1 ± 6.4	168.5 ± 6.9	167.3 ± 6.7	167.1 ± 4.3	0.320
Weight, kg	75.1 ± 5.5	76.1 ± 6.9	74.8 ± 6.3	71.1 ± 5.8	0.563
Smoking, pack-years	37.7 ± 6.5	34.3 ± 6.6	34.2 ± 5.5	36.3 ± 3.5	0.892
Smoking, status					0.426
Current Smoker	25 (59.5)	23 (54.8)	25 (59.5)	28 (66.7)	
Ex-Smoker	17 (40.5)	19 (45.2)	17 (40.5)	14 (33.3)	
GOLD stage					0.328
I	6 (14.3)	6 (14.3)	3 (7.2)	3 (7.1)	
II	18 (42.8)	15 (35.7)	20 (47.6)	15 (35.7)	
III	14 (33.4)	16 (38.1)	18 (42.8)	19 (45.3)	
IV	4 (9.5)	5 (11.9)	1 (2.4)	5 (11.9)	
FVC (% of predicted value)	96.5 ± 6.4	79.1 ± 7.4	86.1 ± 6.9	75.7 ± 6.5	<0.01
FEV_1_ (% of predicted value)	61.2 ± 7.1	45.2 ± 7.1	55.4 ± 6.2	37.8 ± 3.8	<0.01
DLCO (% of predicted value)	72.5 ± 9.1	58.3 ± 6.3	64.2 ± 7.8	51.1 ± 4.2	<0.01

### The Levels of MALAT1, miR-146a, PGE1, and COX2 Were Different Among Different Groups

**FIGURE 1:**
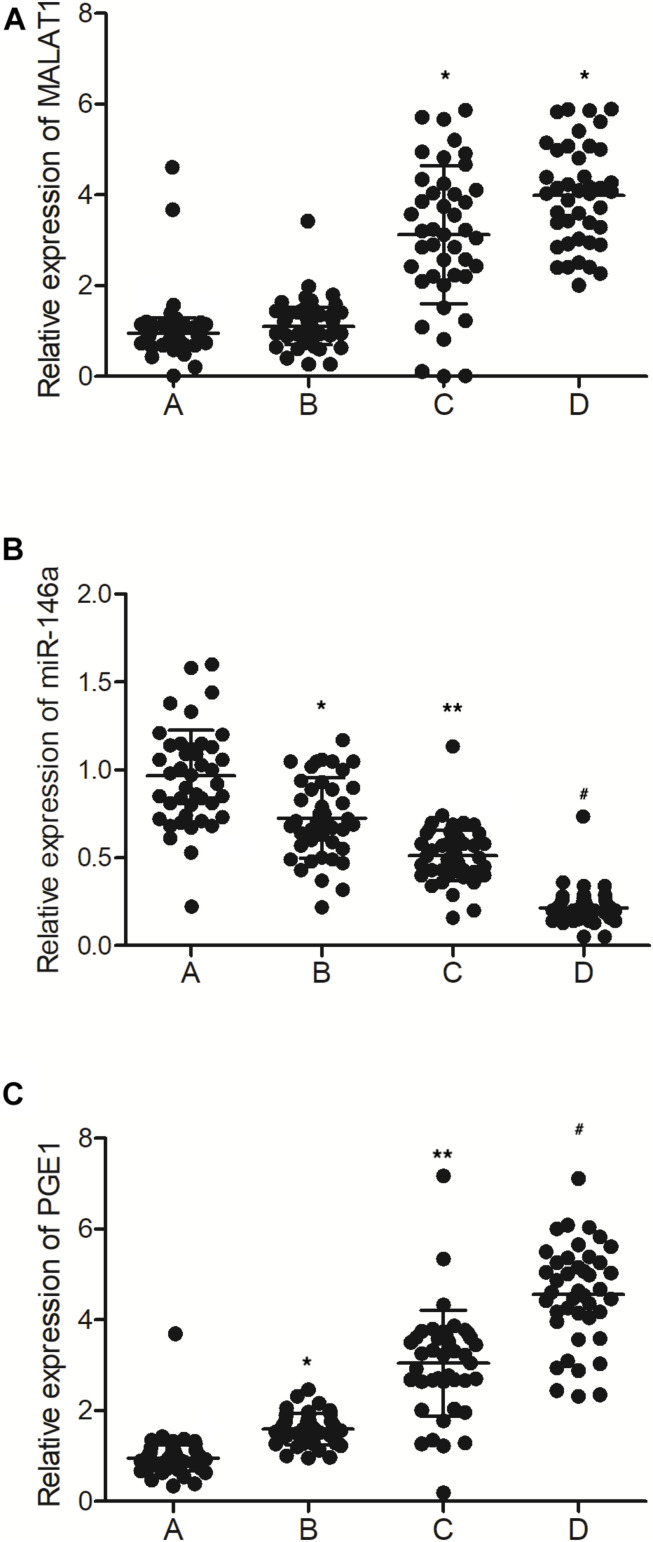
The levels of MALAT1, miR-146a, and PGE1 in peripheral blood samples were different among different groups (A: MALAT1 HYPER + miR-146a HYPO group; B: MALAT1 HYPER + miR-146a HYPER group; C: MALAT1 HYPO + miR-146a HYPO group; D: MALAT1 HYPO + miR-146a HYPER group. *p < 0.05 vs. group A; **p < 0.05 vs. group B; ^#^*p* < 0.05 vs. group C). **(A)** Relative expression of MALAT1 in the peripheral blood samples was similar between the MALAT1 HYPER + miR-146a HYPO group and the MALAT1 HYPER + miR-146a HYPER group, while being lower than that of the MALAT1 HYPO + miR-146a HYPO group and the MALAT1 HYPO + miR-146a HYPER group. **(B)** Relative expression of miR-146a in the peripheral blood samples gradually decreased following the order of MALAT1 HYPER + miR-146a HYPO group, MALAT1 HYPER + miR-146a HYPER group, MALAT1 HYPO + miR-146a HYPO group, and MALAT1 HYPO + miR-146a HYPER group. **(C)** Relative expression of PGE1 in the peripheral blood samples gradually increased following the order of MALAT1 HYPER + miR-146a HYPO group, MALAT1 HYPER + miR-146a HYPER group, MALAT1 HYPO + miR-146a HYPO group, and MALAT1 HYPO + miR-146a HYPER group.

As shown in Figure 1A, the expression of MALAT1 was similar between the MALAT1 HYPER + miR-146a HYPO and MALAT1 HYPER + miR-146a HYPER groups, both of which were lower than that in the MALAT1 HYPO + miR-146a HYPO and MALAT1 HYPO + miR-146a HYPER groups. In addition, the expression of miR-146a (Figure 1B) was the highest in the MALAT1 HYPER + miR-146a HYPO group and the lowest in the MALAT1 HYPO + miR-146a HYPER group. Also, ELISA showed the highest level of PGE1 (Figure 1C) in the MALAT1 HYPO + miR-146a HYPER group and the lowest level of PGE1 in the MALAT1 HYPER + miR-146a HYPO group.

**FIGURE 2:**
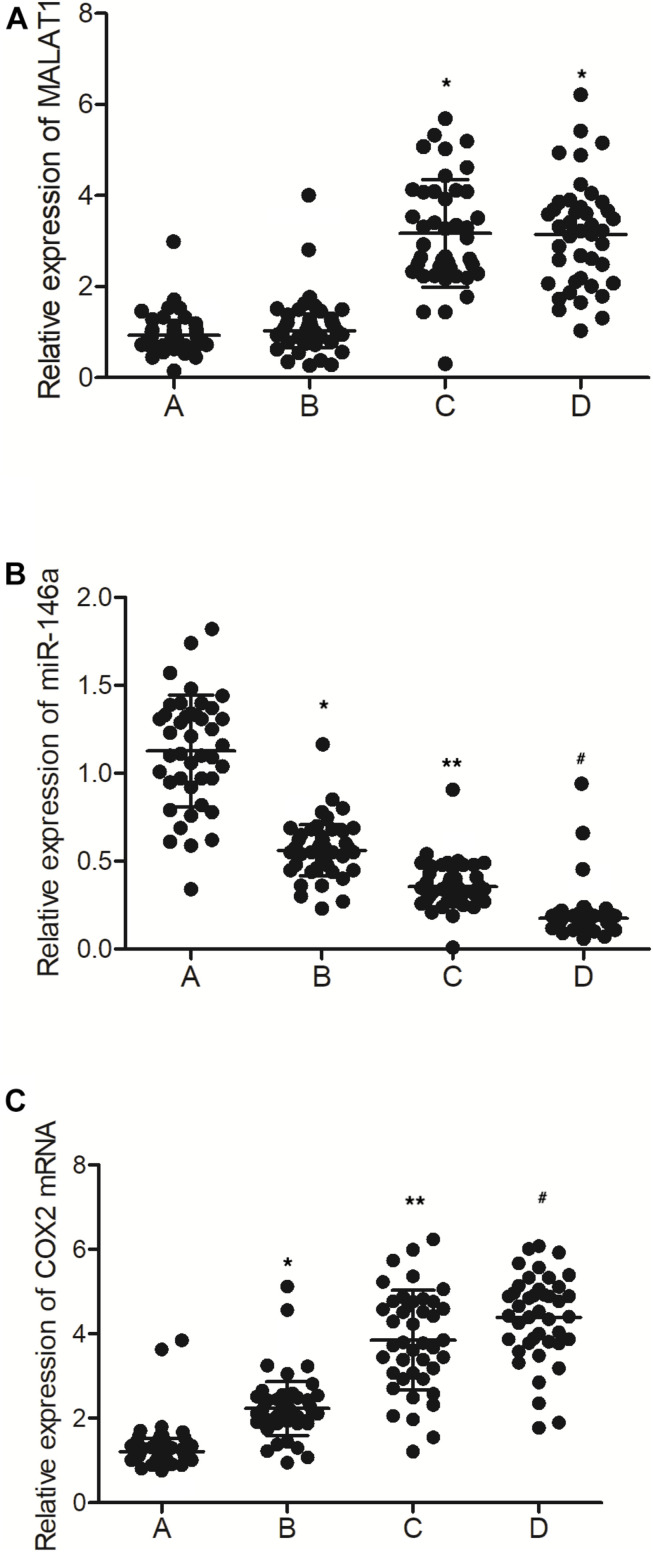
The levels of MALAT1, miR-146a, and COX2 mRNA in PBMC samples were different among different groups (A: MALAT1 HYPER + miR-146a HYPO group; B: MALAT1 HYPER + miR-146a HYPER group; C: MALAT1 HYPO + miR-146a HYPO group; D: MALAT1 HYPO + miR-146a HYPER group. *p < 0.05 vs. group A; **p < 0.05 vs. group B; ^#^*p* < 0.05 vs. group C). **(A)** Relative expression of MALAT1 in the PBMC samples was similar between MALAT1 the HYPER + miR-146a HYPO group and the MALAT1 HYPER + miR-146a HYPER group, while being lower than that of the MALAT1 HYPO + miR-146a HYPO group and the MALAT1 HYPO + miR-146a HYPER group. **(B)** Relative expression of miR-146a in the PBMC samples gradually decreased following the order of MALAT1 HYPER + miR-146a HYPO group, MALAT1 HYPER + miR-146a HYPER group, MALAT1 HYPO + miR-146a HYPO group, and MALAT1 HYPO + miR-146a HYPER group. **(C)** Relative expression of COX2 mRNA in the PBMC samples gradually increased following the order of MALAT1 HYPER + miR-146a HYPO group, MALAT1 HYPER + miR-146a HYPER group, MALAT1 HYPO + miR-146a HYPO group, and MALAT1 HYPO + miR-146a HYPER group.

**FIGURE 3:**
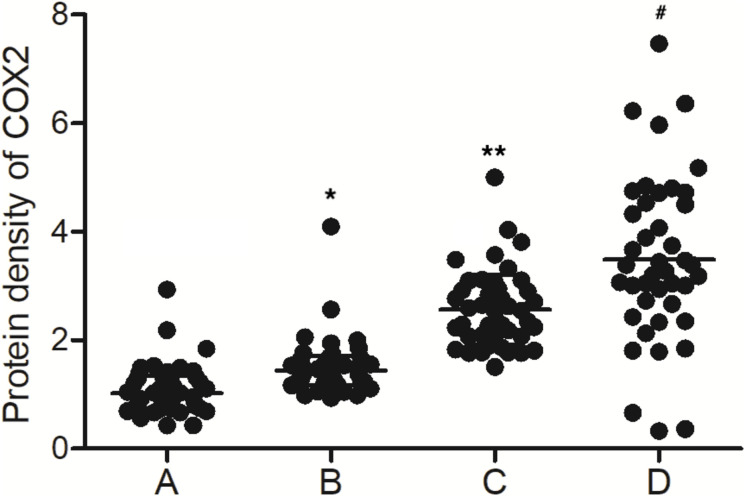
The levels of COX2 protein were different among the MALAT1 HYPER + miR-146a HYPO group, MALAT1 HYPER + miR-146a HYPER group, MALAT1 HYPO + miR-146a HYPO group, and MALAT1 HYPO + miR-146a HYPER group (A: MALAT1 HYPER + miR-146a HYPO group; B: MALAT1 HYPER + miR-146a HYPER group; C: MALAT1 HYPO + miR-146a HYPO group; D: MALAT1 HYPO + miR-146a HYPER group. *p < 0.05 vs. group A; **p < 0.05 vs. group B; ^#^*p* < 0.05 vs. group C).

As shown in Figure 2A, the levels of MALAT1 in the PBMCs of the MALAT1 HYPO + miR-146a HYPO and MALAT1 HYPO + miR-146a HYPER groups were both higher than that in the MALAT1 HYPER + miR-146a HYPO and MALAT1 HYPER + miR-146a HYPER groups. The expression of miR-146a exhibited the same results in PBMC (Figure 2B) and peripheral blood samples (Figure 1B). Moreover, the mRNA (Figure 2C) and protein (Figure 3) expression of COX2 was evaluated in PBMC samples and showed the highest level in the MALAT1 HYPO + miR-146a HYPER group and the lowest level in the MALAT1 HYPER + miR-146a HYPO group.

### Methylation of MALAT1 and miR-146a Promoter Was Different Among Different Groups

**FIGURE 4:**
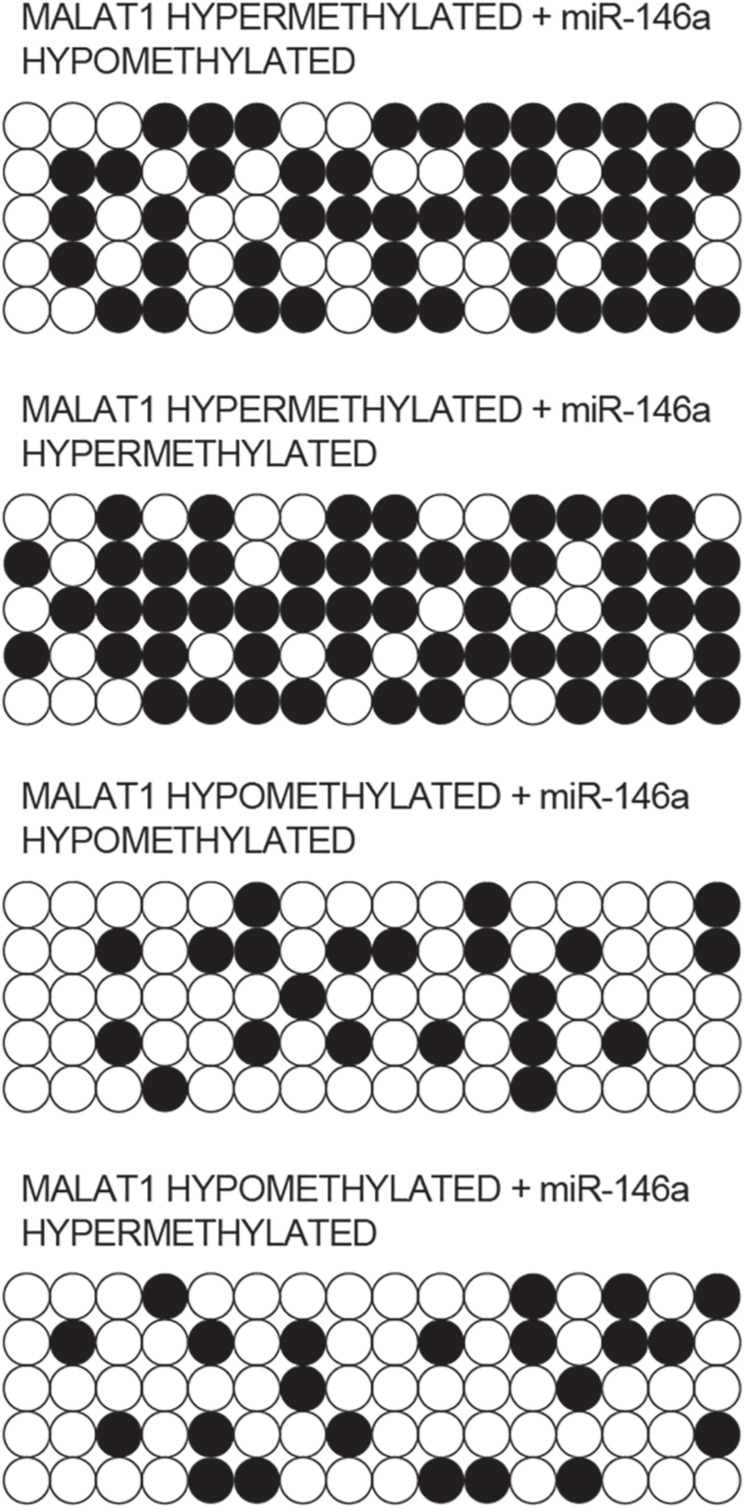
Bisulfite sequencing indicated that the promoter methylation of MALAT1 was comparable between the MALAT1 HYPER + miR-146a HYPO group and the MALAT1 HYPER + miR-146a HYPER group, as well as between the MALAT1 HYPO + miR-146a HYPO group and the MALAT1 HYPO + miR-146a HYPER group.

**FIGURE 5:**
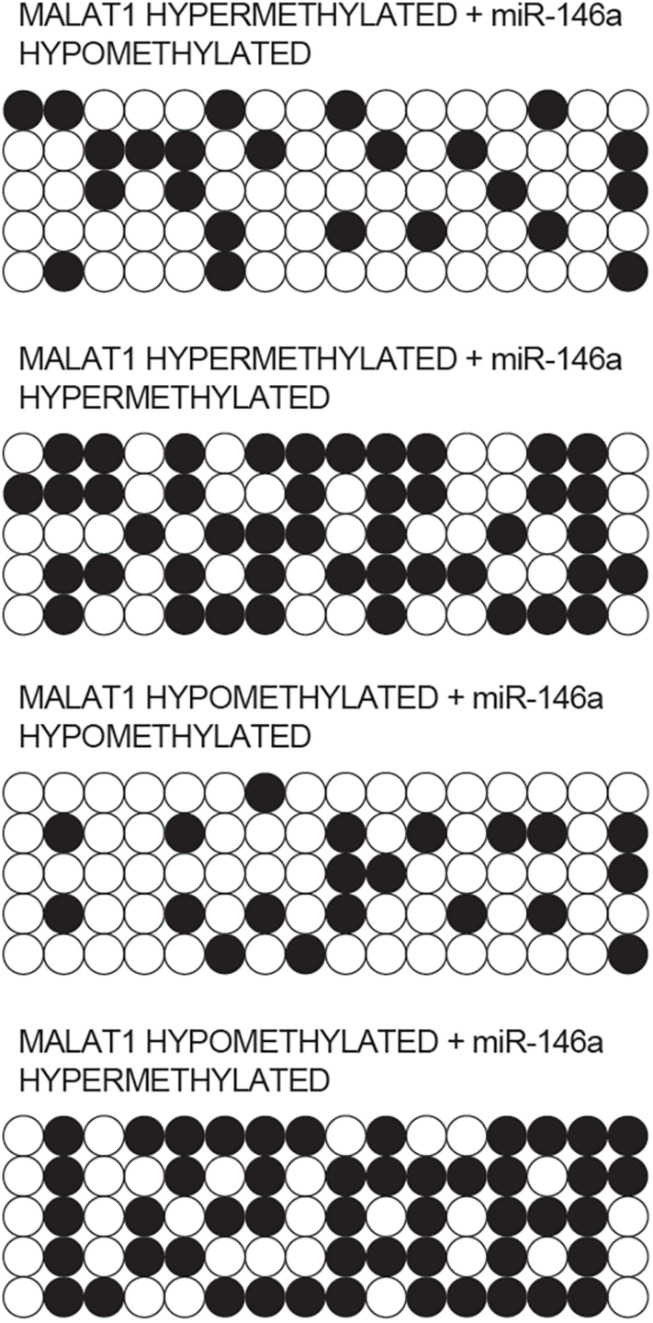
Bisulfite sequencing indicated that the promoter methylation of miR-146a was comparable between the MALAT1 HYPER + miR-146a HYPO group and the MALAT1 HYPO + miR-146a HYPO group, as well as between the MALAT1 HYPER + miR-146a HYPER group and the MALAT1 HYPO + miR-146a HYPER group.

Bisulfite sequencing was conducted to assay the promoter methylation of MALAT1 and miR-146a in each group. As indicated by the results, promoter methylation of MALAT1 (Figure 4) was comparable between the MALAT1 HYPER + miR-146a HYPO and MALAT1 HYPER + miR-146a HYPER groups or between the MALAT1 HYPO + miR-146a HYPO and MALAT1 HYPO + miR-146a HYPER groups. The methylation of MALAT1 was higher in MALAT1 HYPER + miR-146a HYPO and MALAT1 HYPER + miR-146a HYPER groups. Unlike MALAT1, promoter methylation of miR-146a (Figure 5) was comparable between the MALAT1 HYPER + miR-146a HYPER and MALAT1 HYPO + miR-146a HYPER groups or between the MALAT1 HYPER + miR-146a HYPO and MALAT1 HYPO + miR-146a HYPO groups, and the MALAT1 HYPER + miR-146a HYPER and MALAT1 HYPO + miR-146a HYPER groups showed a higher degree of promoter methylation of miR-146a.

### MALAT1 Sponged the Expression of miR-146a

**FIGURE 6:**
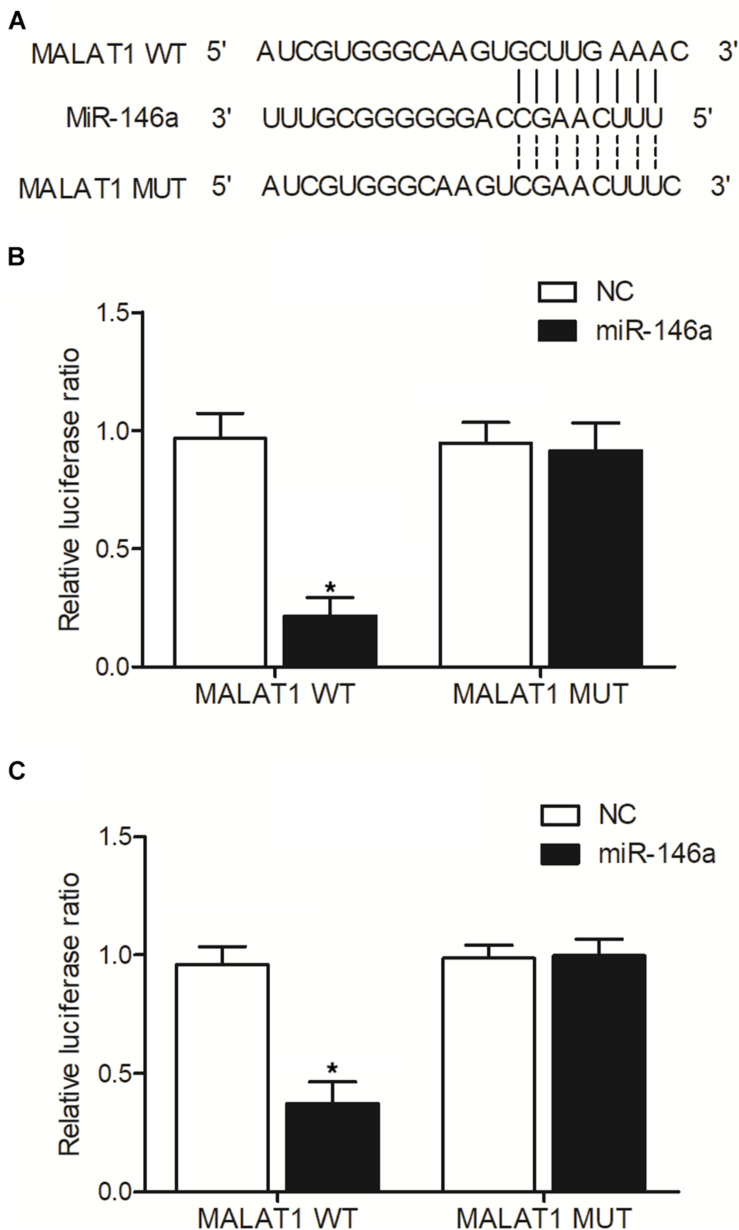
MALAT1 was targeted by miR-146a (*p < 0.05 vs. NC group). **(A)** Computational analysis of MALAT1 and miR-146a sequences. **(B)** Relative luciferase activity was reduced in hPASMCs co-transfected with wild-type MALAT1 and miR-146a. **(C)** Relative luciferase activity was reduced in rPASMCs co-transfected with wild-type MALAT1 and miR-146a.

As indicated by our computational analysis, a putative binding site of miR-146a was identified in MALAT1 (Figure 6A). Luciferase assay was then conducted in hPASMCs and rPASMCs to study the relationship between MALAT1 and miR-146a. As shown in Figure 6B and compared with other groups, the luciferase activity was the lowest in hPASMCs co-transfected with wild-type MALAT1 and miR-146a, indicating MALAT1 as a target gene of miR-146a. Accordingly, similar results were obtained in rPASMCs (Figure 6C). Therefore, it could be validated that MALAT1 was targeted by miR-146a.

### Effect of Upregulation or Downregulation of MALAT1 on the Expression of miR-146a and COX2

**FIGURE 7:**
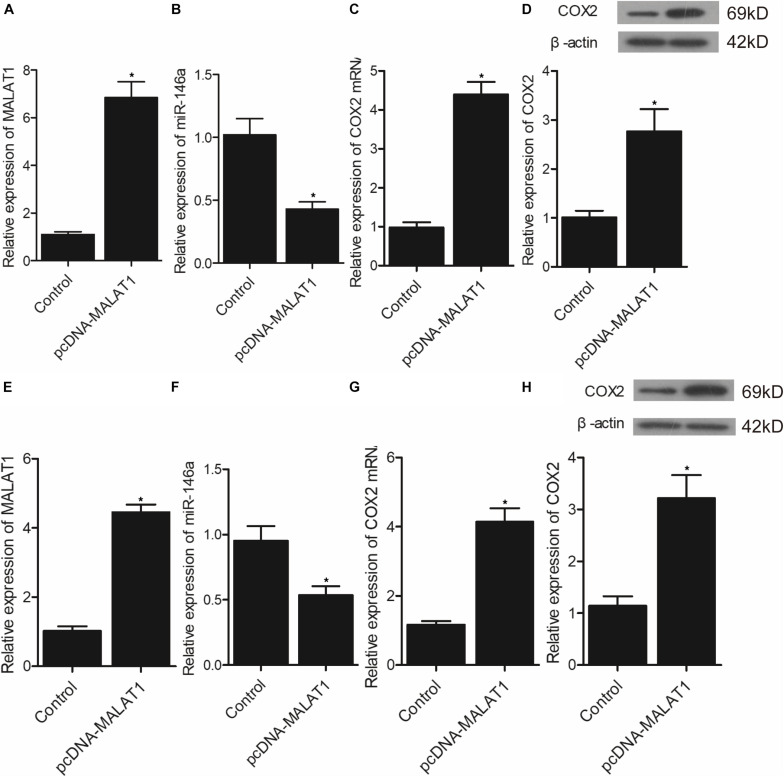
Transfection of pcDNA-MALAT1 inhibited the expression of miR-146a but promoted the expression of COX2 mRNA/protein (*p < 0.05 vs. control group). **(A)** Relative expression of MALAT1 was highly increased in hPASMCs transfected pcDNA-MALAT1. **(B)** Relative expression of miR-146a was suppressed in hPASMCs transfected with pcDNA-MALAT1. **(C)** Relative expression of COX2 mRNA was promoted in hPASMCs transfected with pcDNA-MALAT1. **(D)** Relative expression of COX protein was boosted in hPASMCs transfected with pcDNA-MALAT1. **(E)** Relative expression of MALAT1 was highly increased in rPASMCs transfected with pcDNA-MALAT1. **(F)** Relative expression of miR-146a was suppressed in rPASMCs transfected with pcDNA-MALAT1. **(G)** Relative expression of COX2 mRNA was promoted in rPASMCs transfected with pcDNA-MALAT1. **(H)** Relative expression of COX protein was boosted in rPASMCs transfected with pcDNA-MALAT1.

**FIGURE 8:**
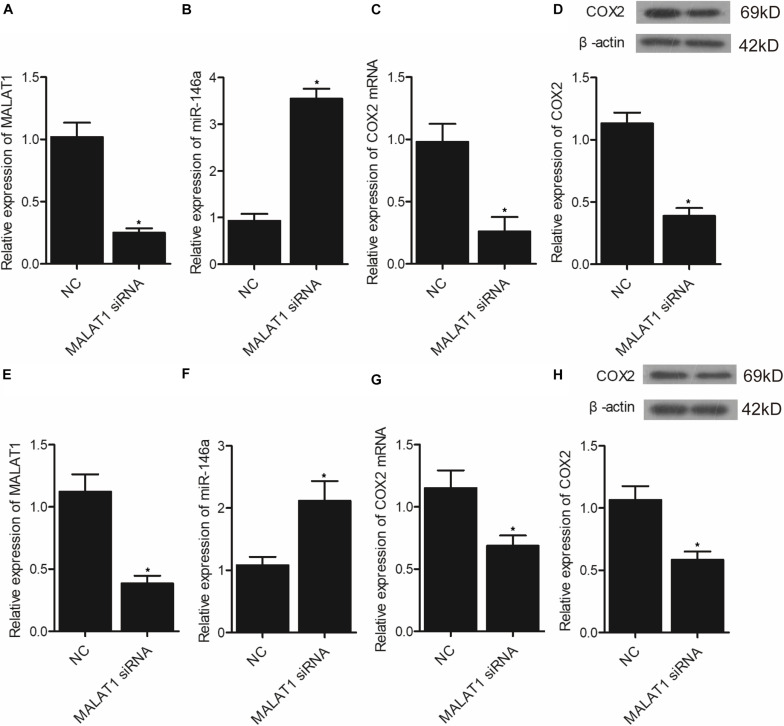
Transfection of MALAT1 siRNA upregulated the expression of miR-146a but downregulated the expression of COX2 mRNA/protein (*p < 0.05 vs. NC group). **(A)** Relative expression of MALAT1 was inhibited in hPASMCs transfected with MALAT1 siRNA. **(B)** Relative expression of miR-146a was increased in hPASMCs transfected with MALAT1 siRNA. **(C)** Relative expression of COX2 mRNA was decreased in hPASMCs transfected with MALAT1 siRNA. **(D)** Relative expression of COX protein was suppressed in hPASMCs transfected with MALAT1 siRNA. **(E)** Relative expression of MALAT1 was inhibited in rPASMCs transfected with MALAT1 siRNA. **(F)** Relative expression of miR-146a was increased in rPASMCs transfected with MALAT1 siRNA. **(G)** Relative expression of COX2 mRNA was decreased in rPASMCs transfected with MALAT1 siRNA. **(H)** Relative expression of COX protein was suppressed in rPASMCs transfected with MALAT1 siRNA.

In addition, real-time PCR and Western-blot analysis were performed to measure the levels of MALAT1, miR-146a, and COX2 in hPASMCs and rPASMCs transfected with pcDNA-MALAT1 or MALAT1 siRNA. As shown in Figure 7, the transfection of pcDNA-MALAT1 significantly enhanced MALAT1 expression (Figure 7A) while reducing the expression of miR-146a (Figure 7B) in hPASMCs. And the mRNA (Figure 7C) and protein (Figure 7D) expression of COX2 was evidently increased in hPASMCs. The above observations were revalidated in rPASMCs (Figures 7E–H). Moreover, the transfection of MALAT1 siRNA significantly reduced the expression of MALAT1 (Figure 8A), as well as the mRNA (Figure 8C) and protein (Figure 8D) levels of COX2 in hPASMCs. And the expression of miR-146a (Figure 8B) in hPASMCs was increased in the presence of MALAT1 siRNA. The same results were found in rPASMCs (Figures 8E–H).

## Discussion

Previous research revealed that miR-146a might be involved in inflammation caused by COPD. In addition, miR-146a could be induced by IL-1b as well as TNF-α, and miR-146a is actually believed to restrict the strength as well as period of inflammatory responses through causing the degeneration of essential mRNAs (Taganov et al., 2006; Togo et al., 2008). Various other researches showed homologies between miR-146a and TNF receptor–associated factor 6 as well as IL-1 receptor–associated kinases. MiR-146a was also negatively associated with the inflammatory responses in lung epithelial cells induced by IL-1b (Taganov et al., 2006; Perry et al., 2008). As a result, the reduced synthesis of miR-146a might additionally contribute to COPD-induced inflammatory responses. FVC, FEV_1_, and DLCO were highest in the MALAT1 HYPO + miR-146a HYPER group and lowest in the MALAT1 HYPER + miR-146a HYPO group. The expression of MALAT1 was similar between the MALAT1 HYPER + miR-146a HYPO and MALAT1 HYPER + miR-146a HYPER groups, both of which were lower than the MALAT1 HYPO + miR-146a HYPO and MALAT1 HYPO + miR-146a HYPER groups. The expression of miR-146a was the highest in the MALAT1 HYPER + miR-146a HYPO group and the lowest in the MALAT1 HYPO + miR-146a HYPER group. Also, both PGE1 and COX2 mRNA/protein expression was highest in MALAT1 HYPO + miR-146a HYPER group and lowest in the MALAT1 HYPER + miR-146a HYPO group.

Cyclooxygenase 2 is actually a rate-limiting enzyme involved in the transformation of arachidonic acid to prostanoids (Herschman, 1996). COX2 is also associated with proliferation as well as growth of various types of cancer cells (Hida et al., 1998). Previous research presented that the incidence of apoptosis, illness, and cancer was associated with the dysregulation in COX2 expression (Scoditti et al., 2012). As an example, reduced COX2 expression dramatically promotes apoptosis induced by genotoxic stress in various types of normal cells (Han et al., 2002). Lots of research highlighted the role of COX2 overexpression in various types of tumors, such as prostate cancer, colorectal cancer, and breast cancer (Fujita et al., 1998). Meanwhile, a number of researches have shown enhanced COX2 expression in the respiratory tracts of COPD patients (Taha et al., 2000; Xaubet et al., 2004). For that reason, COX2 and PGE2 might be primary factors in the pathogenesis of COPD (Taha et al., 2000; Chen et al., 2008). COX2 is actually an immediate gene involved in the early defensive reaction to different stimulations. COX2 is overexpressed in COPD patients (Montuschi et al., 2003; Chen et al., 2008). COX2 regulates *in vivo* synthesis of PGE2, whose level is high in fibroblasts derived from COPD patients, whereas the stimulation by TNF-α and IL-1β, which were actually shown to be related to miR-146a expression, boosted the levels of expression of COX2/PGE2 (Togo et al., 2008; Sato et al., 2010). One research displayed that MALAT1 could regulate COX2 expression by exerting a direct impact on expression of miR-146a (Sato et al., 2010). The results from another research presented that the mean expression of miR-146a varied in subjects with various degrees of severity of silicosis. Similarly, after adjustment for various confounders, smoking and miR-146a expression continued to remain as substantial contributors to silicosis. MiR-146a expression was likewise related to the seriousness of lung dysfunction as well as restricted ventilation, suggesting that miR-146a might be associated with silicosis pathogenesis, as well as its clinical implications (Zhang et al., 2016).

It has actually been noted that lnc-MALAT1 was upregulated in acute respiratory distress syndrome (ARDS), and the high lnc-MALAT1 expression was independently related to a higher risk of ARDS as well as COPD. Furthermore, enhanced expression of lnc-MALAT1 was associated with an elevated level of inflammation, more severe form of the illness, and an increased death rate (Huang and Zhao, 2019). Previous research identified MALAT1 as most significantly dysregulated during hypoxia and showed that MALAT1 expression is actually driven via hypoxia-inducible factor 1α, and the silencing of MALAT1 expression decreases the severity of heart hypertrophy (Brock et al., 2017). Promoter methylation of MALAT was higher in the MALAT1 HYPER + miR-146a HYPO and MALAT1 HYPER + miR-146a HYPER groups, and the promoter methylation of miR-146a was higher in the MALAT1 HYPER + miR-146a HYPER and MALAT1 HYPO + miR-146a HYPER groups. In addition, MALAT1 was targeted by miR-146a, and the transfection of pcDNA-MALAT1 significantly enhanced MALAT1 and COX2 mRNA/protein expression while reducing the expression of miR-146a. Moreover, the transfection of MALAT1 siRNA exhibited an opposite effect compared with the transfection of pcDNA-MALAT1.

This study focused on the methylation on MALAT1 and miR-146a. As MALAT1 and miR-146a are competing endogenous RNAs and may regulate the expression level of the other by sponging each other. Therefore, the deregulation of MALAT1/miR-146a caused by methylation alternation may affect expression of COX2, a direct target of miR-146a. In addition, it has been reported that MALAT1 itself may modify the methylation status of genes (Guo et al., 2015; Biswas et al., 2018; Li H. et al., 2019). Even though there is no evidence that MALAT1 may affect the methylation status of COX2, there is a chance that MALAT1 may indirectly affect the expression of COX2 by modifying some other genes, and this is our future research direction. Moreover, the methylation status of MALAT1 and miR-146a is found to be associated with pulmonary function via regulating expression of COX2, and the methylation status could serve as a predictive and prognostic biomarker of pulmonary function to guide the management of patients with pulmonary diseases.

In conclusion, the findings of this study demonstrated that hypomethylated miR-146a promoter and hypermethylated MALAT1 promoter were associated with milder COPD, an improved pulmonary function, and increased expression of COX2 and PGE1. The methylation status of MALAT1 and miR-146a could be used as a novel biomarker in predicting the severity of COPD.

## Data Availability Statement

The original contributions presented in the study are publicly available. This data can be found here: https://figshare.com/articles/dataset/Original_Source_Date/16554285.

## Ethics Statement

The studies involving human participants were reviewed and approved by Ethics Review Board at Anhui Medical University. The patients/participants provided their written informed consent to participate in this study.

## Author Contributions

LS, WX, and ML was the experimental designer and executor of this study, completing data analysis, and writing the first draft of the manuscript. XX, PL, and KZ were participated in experimental design and analysis of experimental results. GF, SZ, and RW were the designer and leader of the project, guiding experimental design, data analysis, and thesis writing and revision. All authors have read and agreed to the final text.

## Conflict of Interest

The authors declare that the research was conducted in the absence of any commercial or financial relationships that could be construed as a potential conflict of interest.

## Publisher’s Note

All claims expressed in this article are solely those of the authors and do not necessarily represent those of their affiliated organizations, or those of the publisher, the editors and the reviewers. Any product that may be evaluated in this article, or claim that may be made by its manufacturer, is not guaranteed or endorsed by the publisher.
